# Natural deep eutectic solvents in phytonutrient extraction and other applications

**DOI:** 10.3389/fpls.2022.1004332

**Published:** 2022-09-21

**Authors:** Dan Li

**Affiliations:** Botanical Research Lab, Performance Labs Pte. Ltd., Singapore, Singapore

**Keywords:** natural deep eutectic solvent, phytonutrients, enhancing extraction, improving solubility, improving stability, improving oral bioavailability

## Abstract

Natural deep eutectic solvents (NaDESs) are considered a new type of green solvent with attractive application prospects in many fields because of their simple preparation, low cost, environmental friendliness, low volatility, high solvency capacity, designable structure, and easy biodegradability. Due to their biocompatibility, they are safe to use and are particularly suitable for natural product applications. In recent years, NaDESs have been used to extract phytonutrients (e.g., flavonoids, saponins, polysaccharides, alkaloids, quinones, phenolic acids, volatile oils, etc.) to improve their solubility, stability, and bioavailability. This review is intended to summarize and discuss recent progress in the field of natural products related to materials and preparation methods, physicochemical properties, enhancing extraction and separation, increasing solubility, improving stability and bioavailability, facilitating oral absorption of phytonutrients, and finally, highlighting the challenge for future work.

## Introduction

Plants have been supporting life on this planet since the dawn of time. They are essential to human existence, feed us, give us shelter, give the material to transport us, and provide building blocks to make other things like rope, nets, walls, floors, roofs, and more. Plants produce the very oxygen we breathe, and they have been used for medicine since the beginning of time. There are four different types of molecules in plants. These are macronutrients, vitamins, minerals, and a class of compounds called phytonutrients (Erb and Kliebenstein, 2020). Macronutrients (fat, protein, carbohydrates), vitamins, and minerals are all involved to help plant growth in its basic structure. During specific life cycles, the plant starts to produce phytonutrients. These are organic compounds used in defense, protection, signaling, and other undiscovered means. Phytonutrients have beneficial effects on maintaining health and preventing disease. For example, flavonoids contain powerful antioxidants such as catechins and anthocyanins, which are known to help prevent damage to cells throughout the body, and fight against cancer and heart disease (Wen et al., 2021). Phenolics work as antioxidants to reduce inflammation and have been shown to reduce the risk of cancer, heart disease, stroke, Alzheimer’s and Parkinson’s disease (Jacobo-Velázquez and Cisneros-Zevallos, 2017). Estrogen-like phytonutrients can regulate important processes in the skeletal, cardiovascular, and central nervous systems that impact overall health (Kritchevsky, 1997).

One of the challenges is to extract small quantities of secondary metabolites from their respective natural sources. The extraction solvent is a crucial factor in extraction efficiency (Hedrick et al., 1992; Zhang et al., 2012, 2018; da Silva et al., 2016; Kalhor and Ghandi, 2019; Lee et al., 2019; Chandran et al., 2021). Another challenge is to deliver phytonutrients to target tissues and organs for their benefits. The poor water solubility, limited intestinal absorption and low water stability generally lead to nanomolar plasma concentration and lacking pharmacological effect (Shekhawat and Pokharkar, 2017). Tremendous efforts have been made to improve this, such as particle size reduction, solid dispersion formation, surfactant, salt formation, pH adjustment, lipid-based delivery systems, complexation with cyclodextrin, and co-solvent (Bilia et al., 2019; Salehi et al., 2020; Zheng and McClements, 2020; Zuccari et al., 2020). Using non-toxic solvent is one of the simplest and most common approaches. A novel type of green solvent called natural deep eutectic solvents (NaDESs) have gained increasing attention to replace toxic organic solvents for extraction and improving solubility and bioavailability, due to their many advantages such as sustainability, biodegradability, acceptable pharmaceutical toxicity profiles, and high solubilization power of both polar and nonpolar compounds (Dai et al., 2013b; Paiva et al., 2014a; Cunha and Fernandes, 2018; Fernández et al., 2018; Vanda et al., 2018; Liu et al., 2018a; Benvenutti et al., 2019; Choi and Verpoorte, 2019).

NaDESs are a new class of sustainable solvents, often considered renewable, inexpensive and green solvents because they are environmentally friendly solvents derived from the processing of crops that optimize solubility, viscosity, selectivity, and other physicochemical properties for specific applications. A NaDES is a mixture of two or more natural components including sugars, sugar alcohols, polyalcohols, amino acids, organic acids, and organic bases, named hydrogen bond donor (HBD) and hydrogen bond acceptor (HBA). The combinations of two or more of these compounds in specific molar ratios with the addition of water can form an intermolecular hydrogen bonding that lead to charge delocalization, resulting in a mixture with a melting point lower than that of the constituents themselves. Scientists discovered that these liquids are essential for biosynthesis and biochemical processes in living beings, enhancing the solubility of some molecules and promoting chemical reactions (Choi et al., 2011). NaDESs show great potential in the natural product field. They have been applied to enhance extraction, improve solubility, enhance biological activity, and promote oral absorption. This short review summarizes the recent research on NaDESs, their physicochemical properties and applications in the natural products field, and the principles of NaDESs formation and preparation.

## Components and preparation NaDES

**Figure 1:**
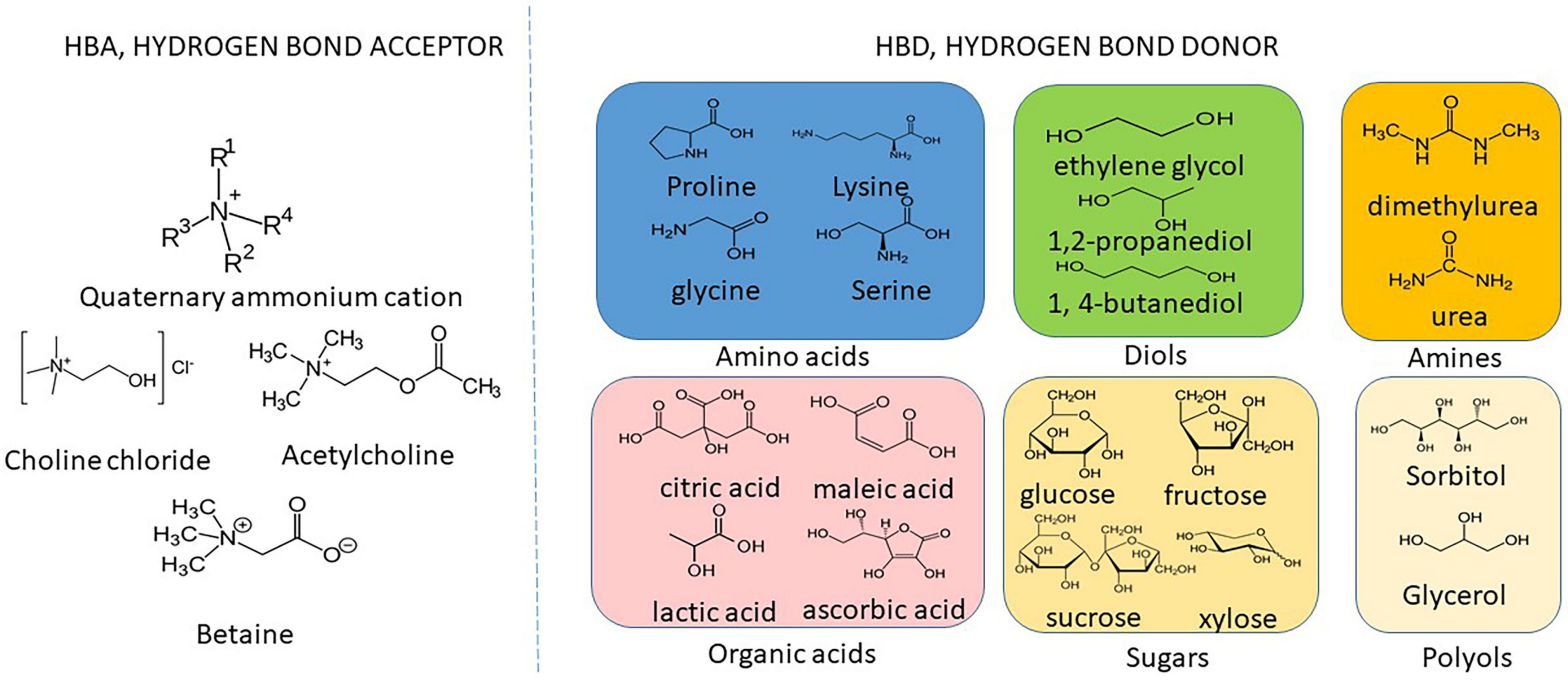
Chemical structures of commonly used HBAs and HBDs for the preparation of NaDESs.

In 2003, Abbott and colleagues reported mixture of urea or organic acids with quaternary ammonium salts could become liquid when heated to 80–100°C. In those liquids, two urea molecules or two carboxylic acid groups were required to complex each chloride ion (Abbott et al., 2003). The resulting liquid, named as deep eutectic solvents (DESs), was found to have exciting solvent properties, similar to those of ionic liquids (ILs). Similar to ILs or DESs, NaDESs are formed by mixing two or more components from natural sources. The common HBAs cover quaternary ammonium salts (choline chloride), amphoteric ions (betaine), and the like. HBD covers organic acids, polyols and sugars that can form hydrogen bonds with the anion in HBA, as shown in Figure 1. Water molecules can be used as one of the components of some NaDESs. For example, Choi et al. (2011) and Francisco et al. (2012) used natural amino acids as HBA and natural phytic acid as HBD to synthesize non-toxic, biodegradable NaDESs.

The formation of NaDESs has closely related to the interactions between components, and the detailed mechanism is still unclear. Most of the views suggest the hydrogen bonding between the anion of halide salt and HBD is the leading force for forming NaDESs. Hydrogen bonds lower the lattice energy of the constituent molecules, resulting in a mixture with a lower melting point and a liquid state (Dai et al., 2013c; Francisco et al., 2013; Liu et al., 2018a). Dai et al. used nuclear magnetic resonance spectroscopy to observe the existence of hydrogen bonds in NaDESs and demonstrate water was also involved in forming NaDESs (Dai et al., 2013b). The influence of the structure of the compounds on the formation and stability of NaDESs was evaluated, and it was found that the number of HBD or HBA, the spatial structure of the functional groups, and the position of the bonds had significant effects.

NaDESs are prepared by heating (Dai et al., 2013b; Liu et al., 2018a; El Achkar et al., 2019; Roda et al., 2019), freeze drying (Gutiérrez et al., 2009; Jeong et al., 2015a; Şahin, 2019), and vacuum evaporation methods (Wikene et al., 2015b, 2016; Liu et al., 2018b). If the components of NaDESs are dry compounds and have good thermal stability, they can be prepared by heating (Gajardo-Parra et al., 2019). The HBD and HBA are mixed in an appropriate molar ratio at a specific temperature (50–100°C) and stirred with a magnetic stirrer until a homogeneous clear liquid forms. If the components of NaDESs are heat-sensitive, they can be prepared by evaporation. The components of NaDESs are dissolved in water, and the water is removed by vacuum evaporation. The freeze-drying method is based on aqueous solutions of individual thermally unstable components. The components are mixed in appropriate molar proportions and dissolved with a small amount of water. The mixture is then freeze-dried for not <24 h until the weight remains constant.

## Physicochemical properties of NaDES

### Phase behaviour

NaDESs are new liquid phase formed by the hydrogen-bonding interaction of two or more solids mixed in a certain mole ratio (Abbott et al., 2001; Smith et al., 2014). For example, choline chloride (melting point, 302°C) and urea (melting point, 133°C) mixed in a 1:2 molar ratio yields a NaDES with a melting point of 12°C (Abbott et al., 2003). Most NaDESs have a melting point below 150°C, and those with a melting point below room temperature have been used as inexpensive and safe solvents in various applications (Zhang et al., 2012; Satlewal et al., 2018). The melting point of NaDESs is related to the hydrogen bond formed between HBD and HBA (Smith et al., 2014; Makoś et al., 2020). The stronger the hydrogen bond, the lower the melting point (Abbott et al., 2003). Abbott et al. found a correlation between melting point and molecular weight of organic acids. The lower the molecular weight, the greater the decrease in melting point (Abbott et al., 2004). Zhang et al. (2012) found that when the molar ratio of choline chloride to urea was 1:1 and 1:2, the melting points of the generated NaDESs were above 50 and 12°C, respectively. Qin et al. (2020) pointed out the molar ratio of HBA to HBD significantly affects the NaDESs. The lowest melting point depends on the nature of HBD.

### Viscosity

NaDESs are highly viscous at room temperature, and their viscosities are mostly between 0.1 and 50  Pa•s, which is 20–1,000 times higher than that of water (Dai et al., 2013a). The viscosity of NaDESs is mainly influenced by Van der Waals forces and hydrogen bonding, and is related to the nature of HBA and HBD, molar ratio, temperature, water content, and more (Abbott et al., 2004; Shah and Mjalli, 2014; Dai et al., 2015; Zhao et al., 2015; Du et al., 2016; El Achkar et al., 2019). Schottky defect, gas-oriented, and cavity theory are commonly used to predict viscosity (Abbott et al., 2006). Abbott et al. used cavity theory to explain the relationship between the viscosity of NaDESs and the mobility of the ions (Abbott et al., 2004). Gajardo-Parra et al. (2019) found that, at below 313 K (39.85°C), the viscosity decreased significantly with increasing temperature, but further increase in temperature resulted in only minor changes in solvent properties. Du et al. reported when water was added to the system, the viscosity decreased significantly. At 25°C, the viscosity of dry choline chloride-urea was 13 times higher than that of hydrated choline chloride-urea (6% water) (Du et al., 2016).

### Surface tension

Similar to high-temperature molten salts and ILs, the surface tension of NaDESs is high, mainly due to intermolecular forces, cation type, temperature and other factors (Vigier et al., 2015; Satlewal et al., 2018). Garcia et al. found that the hydroxyl group in the cation leads to a higher surface tension due to its hydrogen bonding ability. The experimental results showed that increasing the alkyl chain length of the cation leads to higher surface tension (García et al., 2015). The surface tension of glucose-based NaDESs is higher than that of carboxylic acid-based NaDESs (Hayyan A. et al., 2013). AlOmar et al. (2016) found the surface tension of NaDESs decreased with the increase of HBA molar fraction due to the reduction of intermolecular interaction.

### Electrical conductivity

Due to the high viscosity of NaDESs, most NaDESs exhibit a low conductivity, and the magnitude of conductivity is related to temperature and composition of NaDESs (Dai et al., 2013b; Liu et al., 2018a). The conductivity of NaDESs increases significantly with the temperature. This is because the kinetic energy generated by heating increases the frequency of collisions between molecules, weakening intermolecular forces and increasing conductivity (Zhao et al., 2015; Qin et al., 2020). The Arrhenius-like equation can be used to predict the electrical conductivity of NaDESs. Abbott et al. found the conductivity obtained by this equation was linearly related to the inverse of the viscosity of NaDESs (Abbott et al., 2004). Abbott and colleagues also found the conductivity increased with the increase of choline chloride content (Abbott et al., 2007). When the molar fraction of choline chloride increased to 25%, the conductivity of choline chloride/glycerol reached a maximum.

The addition of water increases the NaDESs electrical conductivity. Shah et al. reported when 10% water was added, a choline chloride:Urea eutectic liquid showed a 3-fold increase in conductivity and a more than 80% decrease in viscosity (Shah and Mjalli, 2014). The molar ratio and structure of HBA and HBD had a significant effect on the conductivity of NaDESs. Zhao et al. found electrical conductivity of NaDESs is stronger when NaDESs contain more hydroxyl groups because hydrogen bonding from the hydroxyl group leads to a larger ion mobility (Zhao et al., 2015).

### Solubility

As extractants, NaDESs have good solubility for many components, including natural products, drugs, metal oxides, and carbon dioxide (Nerurkar et al., 2005; Paiva et al., 2014b; Aroso et al., 2016; Li and Lee, 2016; Xie et al., 2016). The solubility of NaDESs can be regulated by changing their components, molar ratio, temperature and water content (Dai et al., 2013b; Cysewski and Jeliński, 2019; Jeliński et al., 2019b). Because of the extensive hydrogen bonding structure in NaDESs, which results in high viscosity and leaves no room for dissolving other solutes, water is needed to disintegrate the bonding structure. Dai et al. investigated the effect of water content on NaDESs and found that adding small amounts of water to NaDESs could increase their solubility (Dai et al., 2013b). This may be related to the change of hydrogen bonding system, but the optimal water content depends on the compound.

The solubility of NaDESs was also found to be greatly influenced by temperature. When the temperature was increased from 40 to 50°C, the solubility of quercetin in glucose/choline chloride increased by 2.3-fold, and the solubility in propylene glycol/choline chloride increased 1.65-fold. Dai et al. proposed the solubility of NaDESs is related to the polarity of the solute. The solubility of non-polar compounds was highest in pure NaDESs, while the solubility of medium-polar compounds was highest in NaDESs containing 5–10% water (Dai et al., 2015).

## Application of NaDES in phytonutrients

### Enhanced extraction and separation

As new green alternatives to traditional solvents, NaDESs have been widely used for the extraction of flavonoids, saponins, polysaccharides, alkaloids, phenolic acids, quinones, volatile oils and other active components from natural sources. The application examples of NaDESs in the extraction phytonutrients are shown in Table 1. It can be seen that the optimized NaDESs are more efficient than the traditional methods for the extraction of the active ingredients. The appropriate amount of water is needed to reduce viscosity and regulate polarity.

**Table 1:** Examples of application of NaDESs in the extraction of phytonutrients from botanicals.

Category	Source material	Active compounds	NaDES and ratio	Water (%)	Results	References
Volatile oil	Cumin	Essential oil	Choline chloride and L-lactic acid (1:3)	40	The samples were pretreated with low eutectic solvents. The extracted essential oils contained more volatile components than those without pretreatment	Zhao et al. (2019)
	*Angelica sinensis radix*	Essential oil ligustilide	Choline chloride–citric acid (1:2)	40	Assisted by microwave hydrodistillation, the extraction yield was improved	Fan and Li (2022)
Terpene lactones	Artemisia	Artemisinin	Methyltrioctylammonium chloride–1-butane alcohol (1:4)		The extraction rate of artemisinin was (7.9936 ± 0.0364) mg/g, which was significantly higher than that of the traditional organic solvent petroleum ether	Cao et al. (2017)
	*Ginkgo biloba*	Terpene lactones	Betaine–ethylene glycol (1:3)	40	Higher terpene lactones than the most efficient solvent (70% ethanol). The total extraction rate was (1.94 ± 0.03) mg g^−1^, with a 99.37% recovery in a single run	Su et al. (2017)
Alkaloids	*Peumus boldus*	Boldine	l-Proline–oxalic acid (1:1)	20	Boldine extraction yield 2.36 mg/g that was 8 times higher than methanol	Torres-Vega et al. (2020)
	*Narcissus pseudonarcissus*	Galanthamine	Malic acid–sucrose (1:1)	50	Similar yield was obtained by both NaDES and methanol, but NaDES showed better selectivity	Rachmaniah et al. (2022)
Flavonoids	*Sophora japonica*	Rutin	Choline chloride–triethylene glycol (1:4)	20	The extraction rate of rutin reached (194.17 ± 2.31) mg/g, showed significant advantages than 60% ethanol and 60% methanol	Zhao et al. (2015)
	*Flos sophorae*	Quercetin, kaempferol Isorhamnetin	L-Proline–glycerol (2:5)	10	NaDESs combined with ultrasound assisted extraction, the extraction rates were 126.7, 3.7 and 13.3 mg/g for quercetin, kaempferol and isorhamnetin, respectively, which is more environmentally friendly than methanol-ultrasonic extraction and thermal reflux extraction	Nam et al. (2015)
	*Radix Scutellariae*	Baicalin, wogonoside, baicalein and wogonin	Choline chloride–lactic acid (1:2)	20	The extraction yield of Baicalin, wogonoside, baicalein and wogonin were(33.10 ± 1.02), (8.32 ± 0.34), (9.21 ± 0.36), (1.637 ± 0.060) mg/g, respectively, higher than 60% ethanol	Wei et al. (2015)
	*Scutellaria baicalensis*	Baicalin	Choline chloride–lactic acid (1:1)	40	The extraction rate of baicalin by NaDESs combined with ultrahigh pressure extraction was 116.8 mg/g, which was higher than that of 70% ethanol and thermal reflux and microwave-assisted extraction	Wang et al. (2018)
	*Ginkgo biloba*	Quercetin, myricetin	Choline chloride–oxalic acid–ethylene glycol (1:1:3)	50	The extraction rates of quercetin and myricetin were 1.40 and 1.11 mg/g, respectively. 104.7% and 90.0%, compared with traditional solvents	Tang et al. (2017)
	*Dalbergia odorifera* T. Chen leaves	Prunetin, tectorigenin, genistein and biochanin A	Choline chloride–levulinic acid (1:2)	10	Compared to water, 50% methanol and methanol, 90% choline chloride/levulinic acid was the most effective solvent for the extraction of polar and non-polar compounds from *Phellodendron* spp.	Li et al. (2016)
	Epimedium	Icariin	Choline chloride, betaine and L proline 43 types	25	L-Proline/1-methylurea (1:1), L-proline/propanedioate (1:1), L-proline/levulinic acid (1:2), betaine/malic acid (1:1), and Choline chloride/N,N′-dimethylurea (1:1) and other 14 NaDESs were more effective than methanol	Duan et al. (2016)
Isoflavone	Kudzu roots and soy molasses	Daidzein, Genistein and Puerarin	Choline chloride–citric acid (1:1)	10, 20, 30	Comparing with methanol extraction, NaDES improved extraction yield, and extracts showed higher antioxidant properties and 20–30% improvement in stability	Duru et al. (2022)
Saponin	Notoginseng	Ginsenoside R1, ginsenoside Rg1, ginsenoside Rb1	Choline chloride, betaine and L proline 43 types	25	The amide NaDESs showed higher extraction rates of saponins than the other NaDESs. The extraction rates of 12 NaDESs were comparable to that of methanol, especially for choline chloride/N,N′-dimethylurea (1:1) and L-proline/1-methylurea (1:1), the extracted saponin content was significantly higher	Duan et al. (2016)
	Ginseng	Ginsenoside	Glycerol–L-proline/sucrose (9:4:1)	33.9	The extraction rate of ginsenoside was (8.16 ± 0.12) mg/g, which is significantly higher than the reported solvents and extraction methods, and did not affect the biological activity of the extracted ginsenosides	Jeong et al. (2015b)
Quinone	Rhubarb	Rhubarb acid, rhododendron, rhubarb phenol Rhododendron methyl ether, Aloe vera rhododendron	Choline chloride, betaine and L proline 43 types	25	The extraction rates of quinone by choline chloride/levulinic acid (1:2), choline chloride/oxalic acid (1:1), betaine/levulinic acid (1:2) and L-proline/levulinic acid (1:2) were similar to those of methanol	Duan et al. (2016)
	*Salvia miltiorrhiza*	Cryptotanshinone, Tanshinone I, Tanshinone Tanshinone IIA	Choline chloride–1,2-butanediol (1:5)	30	The extraction rates of cryptotanshinone, tanshinone I and tanshinone IIA were 0.176, 0.181 and 0.421 mg/g, respectively, using ball mill assisted NaDESs, which is higher than ultrasonic methanol extraction	Wang et al. (2016)
Phenolics	*Salvia miltiorrhiza*	Salvianolic acid B, rosmarinic acid, comfrey acid	Choline chloride, betaine and L proline 43 types	25	Most DESs exhibited higher extraction rates of phenolic acids compared to methanol. The highest extraction rate was obtained for choline chloride/acetamide (1:1)	Duan et al. (2016)
	*Umbelliferum officinale*	Chlorogenic acid, caffeic acid	Tetramethylammonium chloride–urea (1:4)	50	The extraction rates of chlorogenic acid and caffeic acid were 9.35 and 0.31 mg g^−1^, respectively. Compared with the previous study, the extraction rates increased by 177% and 138%, respectively	Park et al. (2014)
	Honeysuckle	Chlorogenic acid, caffeic acid, 3,5-dicaffeic acid Caffeoylquinic acid, 3,4-dicaffe Caffeoylquinic acid, 4,5-dicaffeoylquinic acid	Choline chloride–1,3-butanediol (1:6)	10	NaDESs combined with microwave-assisted extraction yields of chlorogenic acid, caffeic acid, 3,4-dicaffeoylquinic acid, 3,5-dicaffeoylquinic acid and 4,5-dicaffeoylquinic were (26.07 ± 1.25), 0.148 ± 0.007), (0.930 ± 0.018), (23.67 ± 1.08), (0.85 ± 0.38) mg/g, respectively. 1.30, 2.34, 1.31, 1.27, 1.16 times than NaDESs combined with ultrasound-assisted extraction	Peng et al. (2016)
	*Prunella vulgaris*	Rosmarinic acid, Isorosmarinic acid glucoside	Choline chloride–ethylene glycol (1:4)	36	The extraction rates of rosmarinic acid and isorosidic acid glycosides were 3.658 and 1.049 mg/g, respectively, which were higher than the other previously reported extraction methods and solvents	Xia et al. (2015)
	*Thymus serpyllum L*	Gallic acid Caffeic acid Epicatechin Rosmarinic acid Luteolin and Quercetin	L-Proline–glycerine (1:2)	25	NaDES extraction have been efficiently used to recovery polyphenolic antioxidants from Wild thyme herbal dust. It provides a tremendous improvement in polyphenol content and antioxidant activity	Pavlić et al. (2022)
Anthocyanin	*Sour cherry pomace*	Cyanidin 3-rutinoside, Quercetin 3-glucoside, Quercetin 3-rutinoside, Cyanidin 3-sophoroside, cyanidin 3-glucosylrutinoside	Choline chloride–malic acid (1:1)	20	Extract based on ChCl:MalA system was 62.33% more efficient for anthocyanin extraction compared with the conventional solvent. This was because of the stability of flavylium cation in a highly acidic medium	Popovic et al. (2022)
Quinone chalcone Ketone glycosides	*Carthamus tinctorius*	Hydroxy saffron yellow pigment cartormin, saffron glucoside	Proline–malic acid–water (1:1:3), lactic acid–glucose/water (5:1:3)	25	75% PMH (proline/malic acid/water) was the best solvent for hydroxy saffron yellow pigment and the extraction rate was comparable to that of water and 8% higher than 40% ethanol; the extraction rate of cartormin was 14% higher than water and 40% ethanol. LGH (lactic acid/glucose/water) was the best solvent for the extraction of saffron glucoside with 23% higher extraction rate than 40% ethanol	Dai et al. (2013c)
Polysaccharides	Yam	Polysaccharide	Choline chloride–1,4-butanediol (1:4)	32.89	The average extraction rate of polysaccharides was (15.98 ± 0.15)%, which was higher than that of hot water extraction and water-ultrasonic extraction	Zhang and Wang (2017)

Most of the NaDESs reported so far are strongly hydrophilic, and the water has a strong ability to form hydrogen bonds, which can break the hydrogen bonds between NaDESs to varying degrees, making hydrophilic NaDESs unstable in aqueous solutions. Therefore, the application of NaDESs in the separation of active ingredients is scarce. Khezeli et al. reported an ultrasound-assisted liquid–liquid microextraction method (UALLME-DESs) based on NaDESs for ferulic acid, caffeic acid, and cinnamic acid extraction from cinnamon oil (Khezeli et al., 2016). NaDES consisting of choline chloride/ethylene glycol (1:2) was first added to the n-hexane containing cinnamon oil, and then the NaDESs were extracted from cinnamon oil. The extraction was accelerated by ultrasonication, and finally, the phase separation was carried out by centrifugation. The target analytes at trace levels were successfully achieved.

### Improving solubility and stability

NaDESs can dissolve a variety of insoluble bioactive compounds and also improve chemical stability (Liu Y. et al., 2016; Lu et al., 2016; Mbous et al., 2017; Olivares et al., 2018; Araya-Sibaja et al., 2019; Pedro et al., 2019). Curcumin has lipid-lowering, antitumor, anti-inflammatory, choleretic, and antioxidant effects and has therapeutic potential in many diseases. However, curcumin is poorly water-soluble, chemically unstable in alkaline media, and rapidly hydrolyzed and degraded at physiological pH. Curcumin is also a photosensitive compound, which is easily and rapidly photodegraded (Goud et al., 2012; Kharat et al., 2017; Kaur et al., 2021). Therefore, the low oral bioavailability of curcumin limits its clinical efficacy. Jelinski et al. found the solubility of curcumin in NaDESs was much greater than solubility in water (Jeliński et al., 2019a). At room temperature, the amount of curcumin dissolved increased 12,000-fold compared to aqueous solutions. In the stability experiments, NaDESs were found to prevent the photodegradation of curcumin. Wikene et al. found the stability of curcumin in citric acid-sucrose was 2- to 10-fold higher than solutions containing cyclodextrin and 1,300-fold higher than in pH 8 buffers. In addition, compared with preparations containing cyclodextrins and surfactant, the photolytic stabilization of curcumin in citric acid-sucrose was improved by 5.6–10 times (Wikene et al., 2015a).

Similarly, the stability of unstable β-lactam antibiotics (Olivares et al., 2018), aspirin (Lu et al., 2016), salvianolic acid B (Chen et al., 2016), and other phenolic compounds was improved in NaDESs. Dai et al. found safflower natural pigments were more stable in sugar-based NaDES than in water or 40% ethanol solution (Dai et al., 2014). This strong stabilizing ability was attributed to the formation of strong hydrogen bonding interactions between the solute and NaDES molecules. The stabilizing ability of NaDESs can be adjusted by reducing the water content and increasing the viscosity. Therefore, NaDESs is a potentially promising solvent and stabilizer for insoluble compounds formulations.

### Improve oral absorption

NaDESs increase the solubility of phytonutrients through hydrogen bonding to improve the oral bioavailability (Gutiérrez et al., 2019; Pedro et al., 2019). Berberine has many therapeutic potentials, but pharmacokinetics studies have shown that berberine is poorly absorbed orally and rapidly metabolized after oral administration, so its blood concentration is extremely low (Liu C. et al., 2016). Three berberine NaDESs solutions and one berberine aqueous solution were administered to mice at 50 mg/kg dose by gavage. The blood concentration of berberine was determined by LC–MS/MS. The pharmacokinetic analysis showed blood concentration of NaDESs berberine increased by 2–20 times (Sut et al., 2017). The increase in bioavailability was mainly related to the solubilization properties of different NaDESs. Faggian et al. used rutin as a model drug, and sugar, amino acids and organic acids as raw materials to prepare rutin-containing NaDESs (Faggian et al., 2016). The pharmacokinetics of rutin-containing NaDESs were studied and compared with the bioavailability of oral aqueous suspensions. The results revealed the relative bioavailability of rutin in NaDESs was increased by about 100% compared to the aqueous solution. NaDESs can promote the absorption of rutin in the gastrointestinal tract and elevate plasma levels for a longer duration. Chen et al. compared the pharmacokinetics of salvianolic acid B in choline chloride-glycerol and water (Chen et al., 2017). The results suggest that choline chloride-glycerol promotes the absorption of salvianolic acid B by increasing the membrane penetration, and provide a basis for the feasibility of NaDESs as carriers for oral formulations.

## Conclusion and outlook

As a new generation of green solvents and oral bioavailability enhancers, NaDESs have the advantages of high biodegradability, low toxicity, non-combustibility, simple preparation, and low cost, and their physicochemical properties can be regulated by HBD and HBA. NaDESs can be used as solvent to improve extraction yield and as pharmaceutical excipients to improve the solubility, stability, and permeability of active nutraceutical ingredients, thus enhancing their therapeutic effects. However, there are many problems and constraints that need to be further explored.

The viscosity of NaDESs is much higher than that of commonly used traditional solvents. The existing literature uses powdered herbs as the raw material for extraction. Although this can improve the mass transfer efficiency, it also increases the difficulty of subsequent solid–liquid separation and makes the process scale-up difficult. NaDESs can be mixed with a certain proportion of water for viscosity and polarity adjustment. Combining with other advanced techniques is also preferable to enhance process. Recent studies have shown that some NaDESs can dissolve lignocellulose (Sharma and Kumar, 2018), a new way to promote the release of intracellular phytonutrients and improve mass transfer efficiency.

NaDESs have a low vapor pressure and conventional reduced pressure concentration is unsuitable for their recovery. The currently reported recovery methods include macroporous resin adsorption, freeze drying, and solid phase extraction (Jeong et al., 2015b). In comparison, the macroporous resin adsorption method has the advantages of simple operation and low cost, which can enrich and separate the extracts at the same time. However, the subsequent regeneration of the resin is still cumbersome. Therefore, searching for efficient regeneration methods is essential for the further development of NaDESs.

Although the individual compounds that makeup NaDESs are mostly non-toxic and have no impact on the environment, this does not guarantee that the NaDESs from which they are made up will also have the same properties. Studies on NaDESs have focused mainly on applications, and little has been reported on their toxicity. Hayyan et al. found that the cytotoxicity of four commonly used NaDESs made of glycerol, ethylene glycol, triethylene glycol, and urea with choline chloride was much higher than their components (Hayyan et al., 2013a,b). It is evident that NaDESs need to be studied in depth before they can be called truly non-toxic. Most of the NaDESs reported in the literature so far are hydrophilic and difficult to apply in aqueous systems, which significantly limits their practical application. The development of hydrophobic NaDESs can extend the application from non-aqueous systems to aqueous systems. The hydroscopicity of NaDESs needs to be evaluated, as it may affect the stability of the solvent. There is a significant lack of information on the interaction between NaDESs and phytonutrients. It is believed that with continuous research, the above problems will be solved, and NaDES will be more widely used in phytonutrients and benefit to mankind.

## Author contributions

The author confirms being the sole contributor of this work and has approved it for publication.

## Funding

The research was funded by Unicity International Inc., Utah, United States.

## Conflict of interest

The author declares that this study received funding from Unicity International Inc. The funder was not involved in the study design, collection, analysis, interpretation of data, the writing of this article or the decision to submit it for publication.

## Publisher’s note

All claims expressed in this article are solely those of the authors and do not necessarily represent those of their affiliated organizations, or those of the publisher, the editors and the reviewers. Any product that may be evaluated in this article, or claim that may be made by its manufacturer, is not guaranteed or endorsed by the publisher.
